# The modulation of emotional memory consolidation by dream affect

**DOI:** 10.3389/frsle.2023.1239530

**Published:** 2023-10-11

**Authors:** Liëtte du Plessis, Gosia Lipinska

**Affiliations:** Department of Psychology, UCT Sleep Sciences, Applied Cognitive Science and Experimental Neuropsychology Team (ACSENT), University of Cape Town, Cape Town, South Africa

**Keywords:** affect, dreams, emotional memory consolidation, REM sleep, SA-APS

## Abstract

**Introduction:**

Research in the field of cognitive neuroscience has focused on the role of sleep in various neurocognitive processes such as memory consolidation. However, an area that has not been adequately researched is the role of dreaming in this memory process. This study aimed to determine the relationship between affect experienced in dreams and emotional memory consolidation. Considering that REM dreams are laden with emotion and that emotion enhances memory, one possibility is that dream affect could also play a role in emotional memory consolidation. We hypothesised that greater dream-related affect would be associated with greater memory retention of emotional but not neutral information.

**Methods:**

126 healthy participants, aged 18–35, were recruited for the online study, of which 103 participants had valid data (female: *n* =73). On the night of the study, participants viewed a series of pictures from the South African Affective Picture System (SA-APS) in an online session. Afterwards, they verbally recalled as many pictures as possible. The following morning, they were asked to recall any dreams and rate the emotional intensity of their dreams. Participants then again verbally recalled all the pictures that they could remember from the previous night.

**Results:**

Contrary to the prediction, dream-related affect, regardless of valence, did not predict memory consolidation of positive or negative information. Instead, increases in dream-related affect, and especially anxiety were predictive of better memory retention of all information. The findings also showed that an increase in negative affect in dreams predicted better memory retention of negative information.

**Discussion:**

Our results suggest that dream affect is an important modulator of memory consolidation processes occurring during sleep. Furthermore, increased negative affect may indicate which experiences are salient and require consolidation to form long-lasting memories that can guide future behaviour.

**Conclusion:**

These findings have implications for psychiatric disorders, such as major depression, which is characterised by negative affect and increased memory sensitivity to negative stimuli.

## Introduction

While much of sleep and memory research has focused on the consolidation of neutral declarative memory, a growing body of research has investigated sleep's contribution to emotional memory consolidation. Emotional memory refers to memory for information that is valenced and/or arousing (Kensinger and Murray, 2012). According to Heuer and Reisberg (1990), emotional stimuli are remembered better than neutral stimuli in real-life events and laboratory studies. A key explanation for this finding is that emotional memories are considered important for guiding future behaviours (Euston et al., 2012; Brosch et al., 2013). Therefore, if these emotional memories are preferentially consolidated, they are easily accessible when needed in future to guide behaviours or decisions. In contrast, neutral memories tend to fade over time as they are less significant for future use (Williams et al., 2022).

Some studies suggest that this preferential consolidation of emotional memory occurs during sleep (e.g., Wagner et al., 2001; Hu et al., 2006; Payne et al., 2008). According to these studies, emotional memories are more readily consolidated in comparison to neutral memories, and this effect is more pronounced after an interval of sleep as compared to an interval of waking (e.g., Wagner et al., 2001, 2006; Hu et al., 2006; Payne et al., 2008; Nishida et al., 2009). For example, Hu et al. (2006) conducted a study with 14 healthy adult participants. The participants were exposed to the International Affective Picture System (IAPS), an emotional memory task that uses a collection of pictures with standardised emotional ratings. Participants completed the emotional memory task that involved an initial study period, which was followed by a recognition trial (12 h later). The task was administered in two phases: once with 12 h of sleep and once with 12 h of wakefulness. This study concluded that emotional memory consolidation is selectively facilitated by sleep, because emotional stimuli, in comparison to neutral stimuli, were better remembered after this period rather than after waking (Hu et al., 2006).

There is, however, contradicting research and some studies have not demonstrated that effect (e.g., Atienza and Cantero, 2008; Morgenthaler et al., 2014; Tempesta et al., 2017; Bolinger et al., 2018; Jones et al., 2018). Atienza and Cantero (2008) conducted a study with 28 participants and found that although emotional images are better remembered than neutral ones after sleep, arousal played a much stronger role than valence. Bolinger et al. (2018), conducted a study with 16 participants and did not find preferential enhancement of negative stimuli over neutral. Therefore, in a meta-analysis assessing the current literature, Lipinska et al. (2019), found sleep did not preferentially consolidate emotional memory in comparison to waking unless certain methodological criteria were observed. This effect was only seen when studies used free recall of memories rather than recognition measures and also when the memory measures (free recall or recognition) were baseline controlled (i.e., the delayed recall was controlled for initial learning) (Lipinska et al., 2019). Furthermore, this preferential consolidation effect was also found in studies that compared negative vs. neutral information, as well as in studies that compared both positive and negative information combined vs. neutral information.

The contradictory evidence regarding emotional memory consolidation suggests that there is an additional factor explaining these divergent findings that has not been explored. Within the literature, there are some potential explanatory mechanisms. This consolidation effect (emotional over neutral memories) seems to occur when REM sleep is considered. According to Wagner et al. (2001), a preference for the consolidation of emotional texts over neutral texts was only seen after a period of REM sleep. This study recruited 23 participants who were instructed to memorise printed texts (neutral and emotional) and recall the texts after different intervals of sleep (REM vs. NREM sleep). Four years later a follow-up study was done with the same 23 participants and had the same findings (Wagner et al., 2006). In Nishida et al. (2009) study, 31 participants were exposed to negative and neutral images from the IAPS. Participants underwent two study sessions before the memory recognition task, where one group of participants had a 90-min nap between the two study sessions and the second group did not. Those in the nap group showed a selective consolidation benefit for emotional information in comparison to participants in the no-nap group. The study also showed that participants who took a shorter time to achieve REM and spent a longer time in that stage consolidated more emotional memory than neutral memory, in comparison to those who had longer REM latency and shorter REM duration. In a recent meta-analysis of 34 sleep studies, Schäfer et al. (2020), found that greater emotional, in contrast to neutral, memory consolidation was evident in studies when participants experienced REM rather than NREM sleep. REM sleep is a unique sleep stage because, during this stage, individuals are likely to experience dreaming, particularly dreams that are longer and contain emotional content, in comparison to dreams elicited from NREM sleep which are more thought-like and less bizarre (Smith et al., 2004).

While there is abundant and contradictory research on the benefit of sleep for emotional memory consolidation, research on the relationship between dreaming and this kind of sleep-dependent consolidation is sparse. Furthermore, dream and emotional memory consolidation associations may help to explain the contradictions in the literature. Some studies have loosely speculated about the link between sleep-dependent memory processing and emotions, imagery and thoughts experienced in dreams (e.g., Payne and Nadel, 2004; Ji and Wilson, 2007). Ji and Wilson (2007) theorised that the reactivation of memories seen in the sensory cortex during sleep might be related to the imagery experienced during dreaming. However, few studies have considered the subjective experience of dreaming and its relationship to emotional memory consolidation (Wamsley and Stickgold, 2011). One such study has found that the incorporation of a recent learning experience (i.e., a maze learning task) into a dream, resulted in better memory performance following a nap (Wamsley et al., 2010). This same study was replicated, and similar results were found following a full-night sleep (Wamsley and Stickgold, 2019). These two studies have established the association between dream content and memory consolidation. However, dreams are not only comprised of content but are laden with emotion. Since dreams, and especially those emerging from the second half of the night and REM sleep, are often highly emotionally charged and contain fragments of recent waking-life experiences (Fosse et al., 2003; Simor et al., 2023), one speculative possibility is that the reactivation of these waking experiences paired together with emotional valence experienced in the dream helps to strengthen the consolidation process. Findings show that affect enhances memory encoding and therefore results in better retrieval during waking life, therefore, if emotions are activated during dreaming, they may also enhance memory consolidation. Apart from this reasoning, there is another related strand of reasoning as to why affect in dreaming may enhance emotional memory consolidation. Many researchers posit that dreams are there to solve emotional problems to guide future behaviour (Cartwright, 1974; van der Helm and Walker, 2011; Lipinska et al., 2019). Therefore, considering that both emotional memory consolidation and dreaming likely exist to solve emotional problems and guide future behaviours, affect in dreaming may modulate emotional memory consolidation to achieve this goal.

Despite there being a robust explanation for why dreams may be linked to memory consolidation, research that attempts to address this hypothesis is lacking (Wamsley and Stickgold, 2011). Our study tests whether affect in dreams will modulate emotional memory consolidation, where affect refers to the subjective experience of emotions.

This hypothesis was tested: the greater the intensity of affect in a dream (regardless of the valence), the greater the memory retention will be for emotional information, but not neutral information, where affect intensity refers to the degree to which a specific affect is elicited rather than the valence or arousal properties of the affect.

However, since the majority of the literature on sleep-dependent memory consolidation compares memory for negative compared to neutral stimuli, with a sparsity of studies examining both negative-neutral and positive-neutral comparisons and that negative and positive stimuli may result in distinct consolidation profiles (Sawangjit et al., 2013), we will also explore the valence-specific association between dream affect intensity and emotional memory retention.

## Methods

### Design and setting

This quasi-experimental study used a virtual-contact study design. Participants completed all study procedures at home and all memory and dream report tasks online with a researcher present virtually.

### Participants

#### Recruitment

We conducted a power analysis to determine the sample size. To achieve a statistical power of 0.8, an average effect size of 0.34 based on previously published studies (Hu et al., 2006; Payne et al., 2008), and to ensure that the predictors are significant, we determined that 113 participants must be involved in the study. We included several control variables in our power analysis, including caffeine intake, smoking, age, gender, and sleep quality (i.e., total sleep time, sleep latency quality, number of awakenings, length of awakenings, and alertness upon waking).

We recruited student participants via the Psychology Department and university-wide networks. Overall, 444 potential participants completed the screening questionnaire, 153 qualified for the study and 126 agreed to participate.

#### Eligibility criteria

Participants were included in the study if they reported: (a) being aged 18–35 years old, (b) not suffering from a sleep disorder (e.g., insomnia) or psychiatric disorder (e.g., PTSD, major depression or substance use disorder), (c) not taking medication for such disorders, (d) being regular sleepers, sleeping between 7 and 9 h a night, (e) not suffering from any medical or neurological condition (e.g., head injury) known to influence sleeping, dreaming or emotional and cognitive functioning, (f) having access to a laptop/computer, phone and a stable internet connexion. Criteria (a)–(e) are all known to influence, sleep, emotional memory processing or dreaming (Krystal, 2012; Cherdieu et al., 2014; Park et al., 2015; Conte et al., 2021; Mayer et al., 2021).

Twenty-three participants were excluded for the following reasons: 20 were excluded for not reporting a dream in the morning. A dream was defined as a long and bizarre story, an image that vanishes rapidly, or waking up thinking of the previous night's pictures they saw. Another three participants were excluded for caffeine intake after 10 am. The final sample size was 103 participants (female: *n* = 73).

### Materials and measures

#### Screening measures

We screened potential participants using an online survey that included standard self-report measures of sleep quality, depression, posttraumatic stress symptoms and alcohol and drug use disorder symptoms. We focused on these specific psychiatric disturbances because they are the most prevalent among young adults in South Africa (Herman et al., 2009). We also collected demographic (e.g., name, surname, gender, age, contact details etc.), medical (e.g., medical conditions, medication, head injury), and sleep-related information (e.g., frequency of dreaming, sleep disorders, approximate average sleep duration).

Regarding sleep quality, the Pittsburgh Sleep Quality Index (PSQI, Smyth, 2000) was used to assess potential participants' sleep quality in the past month. Participants with a global score of > 5 were excluded from participation, based on the measure's clinical cut-off. We used the Patient Health Questionnaire for Depression-9 (PHQ-9, Kroenke et al., 2001) to assess for depressive symptoms. Participants with a score > 9 were excluded from the study because this score indicates the cut-off for mild depressive symptoms. Regarding posttraumatic symptoms, the 5-Item Primary Care Post-Traumatic Stress Disorder Screen (PC-PTSD-5, Prins et al., 2016) was used to characterise these symptoms. Potential participants who answered “yes” to three or more of the questions were excluded from the study. We used the Alcohol Use Disorders Identification Test Consumption (AUDIT-C, World Health Organisation, 2001) to identify and exclude individuals with significant alcohol use disorder symptoms. Women were excluded with scores >5 and men were excluded with scores >7, based on a study conducted in young adult students (DeMartini and Carey, 2012). Regarding drug dependence, we used the Drug Abuse Screening Test (DAST-10, Skinner, 1982) to exclude participants with significant dependence. Participants who answered “yes” to three or more questions were not included in the study.

#### Experimental measures

To evaluate participants' sleep quality, regarding the presence of sleep deprivation or any other disturbances, we used the National Sleep Foundation's sleep diary (National Sleep Foundation, 2021). The sleep diary provided participants' sleep onset latency, sleep efficiency, duration and frequency of awakenings and energy upon awakening.

To test emotional memory we used a South African version of the International Affective Picture System, which is normed for both low- and high-income groups (South African Affective Picture System (SA-APS), Nestadt and Kantor, 2015). Pictorial stimuli fall into three categories, “positive”, “negative”, and “neutral”, according to standardised self-assessment manikin ratings (Bradley and Lang, 1994). The SA-APS consists of 340 pictures and for this study, a series of 72 pictures were chosen for the emotional memory task (i.e., 24 pictures from each valence category). An additional four neutral pictures were used (two at the start of the slideshow and two at the end of the slideshow) to account for any primacy and recency effects. If participants recalled any of these four pictures, it was not considered part of their memory retention score (Ackermann et al., 2015). The selection of pictorial stimuli and the creation of the task is detailed in the Supplementary material. Pictures were presented in randomised order, but no more than four pictures from the same valence were shown consecutively. One black slide and one black slide with a white fixation cross were shown for 500 ms each before a new picture was presented for 2.5 s, following a previously published study (Ackermann et al., 2015). After the participants watched the entire slideshow, they were instructed to verbally recall as many pictures as possible by describing each picture in as much detail as possible. Participants were prompted with phrases such as “Is there anything else you can remember?”. The task did not have a time limit and participants were also not told how many pictures there were.

#### Oral dream report recording and affect rating

Upon waking, participants were asked over the phone to give a spontaneous report of their dreams, according to a standard dream recall protocol (Cartwright et al., 1998). Participants were probed with a question such as “What was going through your mind just before you woke up?” and more directly if their answer was vague, “What did you dream about?” Spontaneous home dream-recall reports have been used in other studies (Blagrove et al., 2011). After recalling their dreams, participants were asked to subjectively rate, on a scale of zero to 10, the intensity of each of the seven specific affects they might have experienced in their dreams, allowing for a total score of zero to 70. The seven affects were based on Panksepp's theory of basic emotions (Panksepp, 1992). These include anticipation/seeking, anger/rage, anxiety/fear, sexual desire/lust, nurturance/care, grief, and joy/play (Van Der Westhuizen and Solms, 2015).

### Procedure and data collection

#### Screening and introductory meeting

Potential participants completed the online screening procedure and eligible participants were consented and invited to attend a 10-min online introductory meeting. During this meeting, the researcher explained study procedures and participants were instructed to abstain from alcohol and regular caffeine consumers were asked to limit caffeine intake to one caffeinated drink (coffee, tea, etc.) before 10 am. Regular smokers continued smoking, but those with occasional smoking habits (e.g., weekend social smokers) were asked to not smoke on the day of the study. Participants were required to complete a sleep diary for at least three days before their scheduled date, including on the night of the study.

#### Night-time procedure

On the experimental study night, the participant and researcher logged onto a Microsoft Teams (MS) meeting half an hour before the participant's usual bedtime. Before starting the emotional memory task, the researcher checked that the participant had set their alarm to their ordinary waking time, 7–9 h from their usual bedtime.

Thereafter the researcher guided the participant through the emotional memory task, which involved watching and recalling the 74 positive, negative and neutral pictures. Once the task ended, participants were reminded to not do anything such as read or spend time on their phones after the meeting ended. They were instructed to go to sleep as soon as possible. Participants were also reminded about the morning procedure where they would have to recall as many pictures as possible from the previous night. This was also outlined during the introductory meeting.

#### Morning procedure

The following morning, the researcher phoned the participant at their awakening time, gave the participant some time to consider their response and collected a dream report, which was recorded. Participants then verbally rated, on a scale from 0 to 10, the following affects experienced in their dreams: anticipation, anger, anxiety, sexual desire, nurturance, grief, and joy.

After providing dream reports, participants completed the emotional memory task again, but without watching the stimuli again. They were instructed to recall as many pictures as they could from the previous night, including any new pictures that they might have remembered and any pictures that they already mentioned the previous night. Thereafter, participants were reminded to submit their sleep diaries. Approximately 1–3 days later, the researcher debriefed the participant telephonically.

### Data analysis

IBM SPSS Statistics Package (version 28.0; IMB Corp, 2021) and Microsoft Excel were used to analyse the data. The alpha level was set to *p* < 0.05.

#### Calculating dream affect

“Total Emotions” was calculated as the sum of the scores for each of the seven affects. For secondary analyses, “Positive Emotions” and “Negative Emotions” were calculated as the sum of the positive emotion scores (seeking, lust, care and play) and negative emotion scores (rage, anxiety and grief), respectively. Both these variables were averaged by the number of affects in each variable, to scale the data for comparison.

Because of violations of the assumptions necessary for parametric data, comparison tests for nonparametric data, such as Wilcoxon signed-rank tests and Friedman tests for multiple comparisons, were run to describe the differences in average dream affects. Bonferroni adjustments were made to prevent type I errors.

#### Calculating memory retention

Both an independent rater and LD determined whether a picture was correctly described or not. Any differences were discussed until a conclusion was reached. The total number of positive, negative, and neutral pictures correctly recalled in each test session was calculated and memory retention scores for each participant were calculated by dividing their morning memory recall by the night memory recall and then expressed as a percentage for each valence category (Positive Retention, Negative Retention, Neutral Retention). An overall memory retention score was also calculated (Total Retention).

A repeated measures ANOVA analysis described the differences in average memory retention across valence categories. Bonferroni adjustments were made to prevent type I errors.

#### Hypothesis testing: predicting memory retention using dream affect

We conducted three primary GLMs to determine whether dream affect (Total Emotions) predicted each Positive, Negative and Neutral Retention, while controlling for age, gender, caffeine intake, smoking, and sleep quality. We undertook a preliminary process of sleep variable selection, to determine which variables would be included in the GLMs. Sleep quality consists of five separate components garnered from participant sleep diaries. These include total sleep time (in hours), sleep latency quality which refers to the ease with which a participant fell asleep, number of awakenings throughout the night, the total length of these awakenings (in minutes), and alertness which refers to how awake the participant felt upon waking. Separate correlation analyses were run on the four memory retention scores (i.e., Total, Positive, Negative and Neutral Retention) and each of the five sleep quality variables. Only the sleep quality variables that had a significant correlation with memory retention scores were included in the GLMs to avoid running many models with multiple terms and thereby increasing type I error probability. We conducted a fourth GLM with Total Retention as an outcome to explore the influence of dream affect on general memory performance. GLMs that did not reveal any significant relationships between dream affect intensity (Total Emotions) and memory retention (Positive, Negative and Neutral Retention) are not reported but can be found in the Supplementary material.

#### Exploratory analyses: investigating the independent contributions of positive and negative dream affects on memory retention

Contrary to our hypothesis, but based on empirical evidence, there is a possibility that positive, negative, or specific dream affects may differentially modulate emotional memory consolidation (Tyng et al., 2017; Williams et al., 2022). Therefore, GLMs that controlled for the same potential confounds as in the previous analysis, examined whether (a) positive and/or negative and (b) each of the individual seven dream affects, predicted memory retention scores for positive, negative, neutral, and total retention with a total of eight general linear models generated.

## Results

### Descriptive statistics

#### Sample characteristics

Table 1 shows the frequencies of participants' self-reported gender, caffeine intake, age, and smoking frequency within the sample.

**Table 1:** Frequency of scores for sample characteristics.

**Variable**	**Frequencies (%)**
**Participant gender**	
Female	73 (70.87%)
Male	30 (29.13%)
**Caffeine intake**	
None	80 (77.67%)
Morning coffee	23 (22.33%)
**Age**	
18–25 years	93 (90.29%)
26–35 years	10 (9.71%)
**Smoking**	
Smoker	8 (7.77%)
Non-smoker	95 (92.23%)

Percentages are reported in parentheses. Caffeine intake indicates use on the day of the study. A participant was classified as a smoker if they reported any use of cigarettes or vaping that involved nicotine.

#### Describing participants' sleep quality

Table 2 shows the self-reported sleep diary data reflecting participants' sleep on the night of the study. The majority of participants managed to sleep 7–9 h (92.23%), however, 7.77% of participants either struggled to fall asleep or woke up before their scheduled alarm and ended up sleeping for <7 h, with the shortest sleep duration reported as 5 h. Sleep latency quality; night-time awakening frequency and length; and morning alertness are also reported in Table 2.

**Table 2:** Frequency of scores for sample sleep quality.

**Variable**	**Frequencies (%)**
**Total sleep time (hours)**	
< 7	8 (7.77%)
7–9	95 (92.23%)
>9	0 (00.00%)
**Sleep latency quality**	
With difficulty	18 (17.48%)
After some time	34 (33.01%)
Easily	51 (49.51%)
**Number of awakenings**	
0	59 (57.28%)
1	24 (23.30%)
2	15 (14.56%)
3	5 (4.85%)
**Length of awakenings (min)**	
0	59 (57.28%)
1–5	22 (21.36%)
6–10	8 (7.77%)
11–20	8 (7.77%)
>20	6 (5.83%)
**Alertness**	
Still tired	24 (23.30%)
Somewhat awake	53 (51.46%)
Wide awake	26 (25.24%)

Percentages are reported in parentheses.

#### Describing participants' dream affect

**Figure 1:**
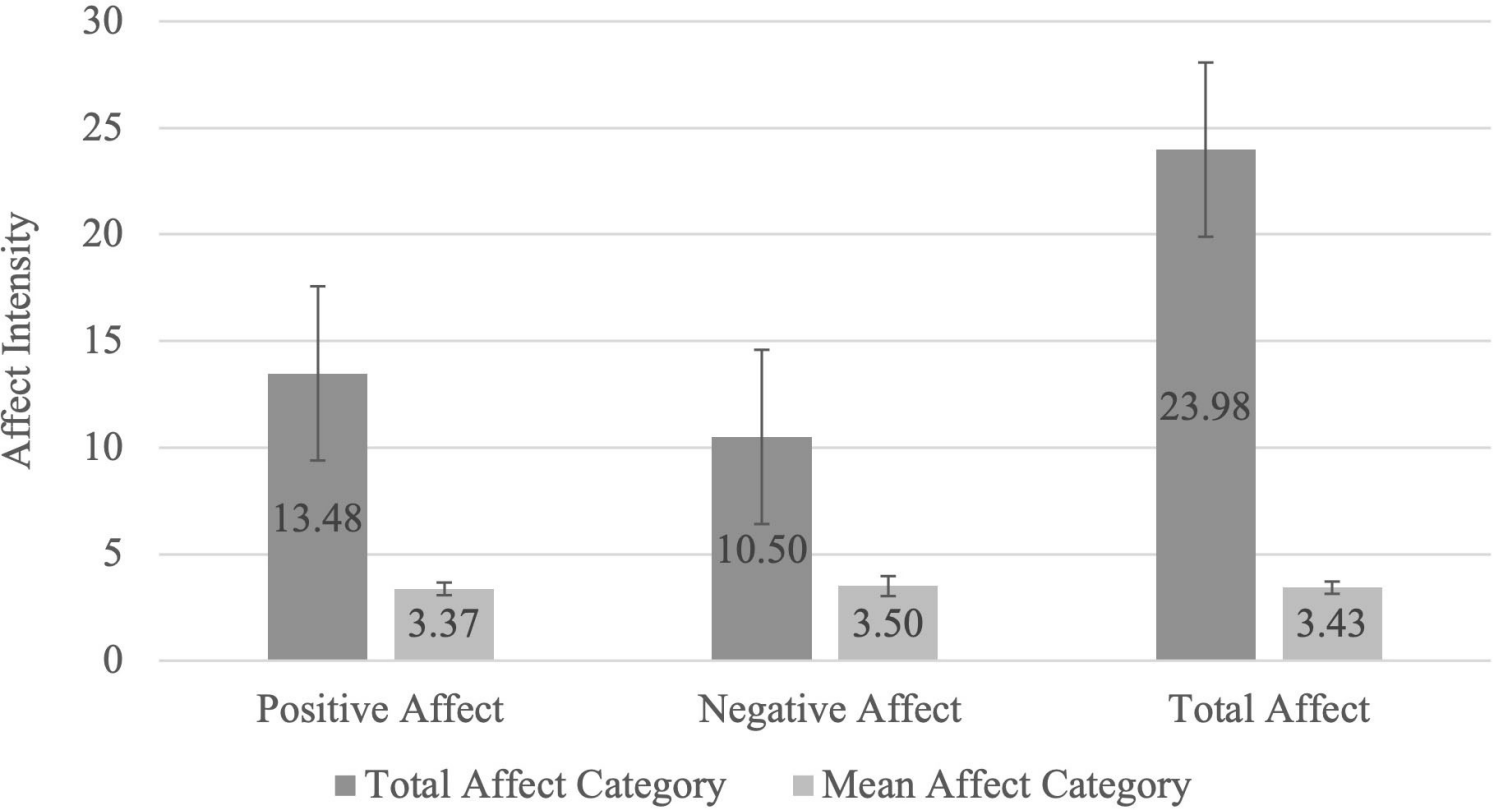
Total and Mean Dream-Related Affects. The Total Affect Category represents the mean affect score for the sum of scores in each affect category (Positive: seeking, lust, care, and play, Negative: rage, anxiety and grief and Total Affect: all seven affects). The Mean Affect Category was determined by dividing the sum of the scores of each affect category (Positive, Negative, and Total Affect) by the number of affects within each category to scale the data for comparison: Positive Mean Affect = Total Positive Affect/4; Negative Mean Affect = Total Negative Affect/3; Total Mean Affect = Total Affect/7.

Figure 1 shows that participants reported an approximately equivalent intensity of positive affects compared to negative affects in their dreams when the number of affects reported was taken into account. A Wilcoxon signed-rank test showed that this difference in average intensity of reported affects is non-significant, with a small effect size (*Z* = −0.578, *p* = 0.563, *r* = −0.057).

**Figure 2:**
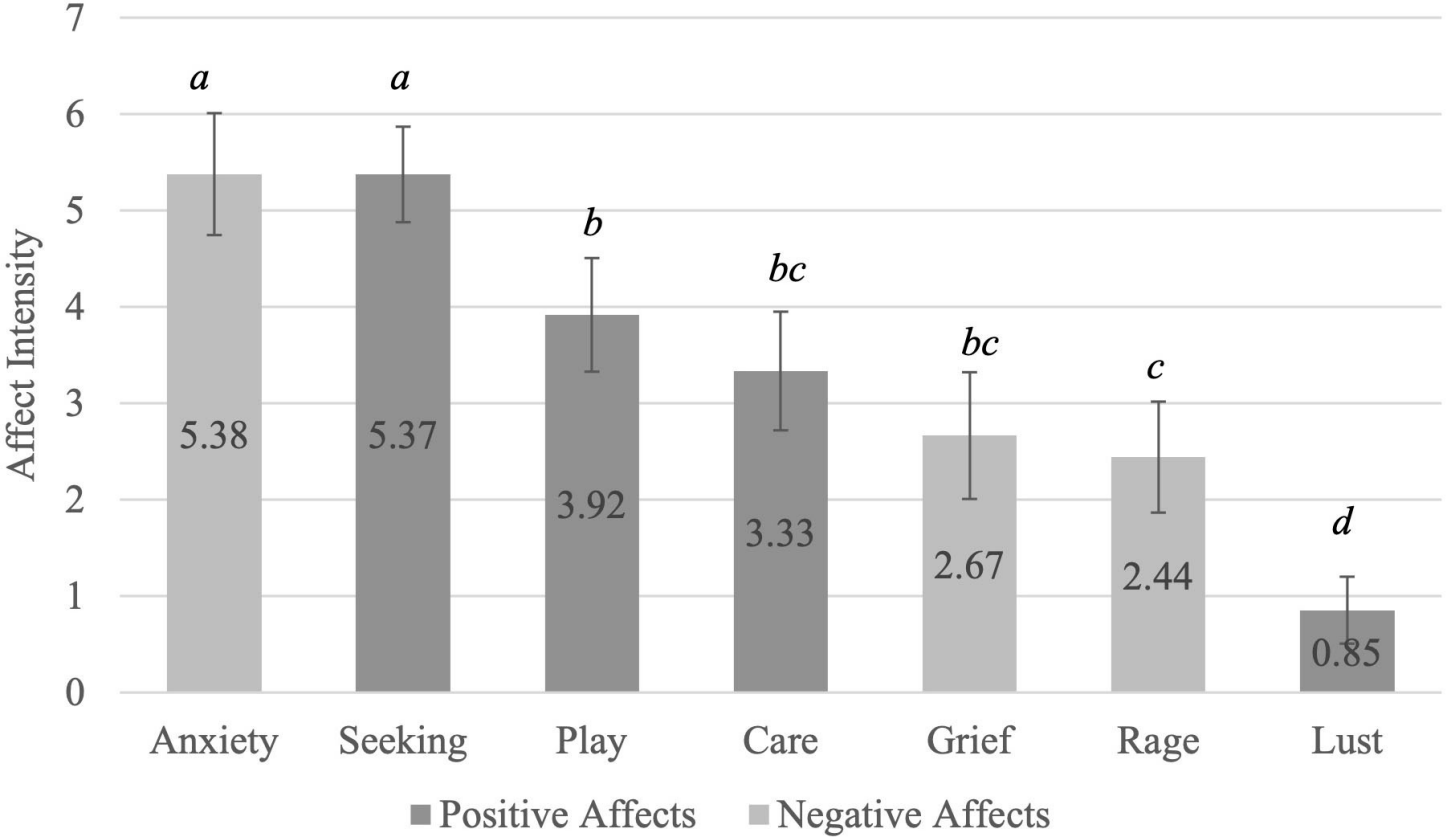
Average Individual Affects Reported in Dreams. Affects with a letter in common are not significantly different from each other. ^a^Anxiety ~ Seeking > All other affects; ^b^Play ~ Care ~ Grief> Lust; ^c^Care ~ Grief ~ Rage> Lust; ^d^Lust < All other affects. Bonferroni adjustments were made due to multiple comparisons. The adjusted α = 0.007.

Figure 2 shows the intensity of the individual affects that participants reported in their dreams. Anxiety was the highest reported affect, followed by anticipation/seeking. The affect with the lowest average intensity reported in dreams is sexual desire. A Friedman test showed that there is a significant difference between the individual affects reported χ^2^(6) = 175.74, *p* ≤ 0.001. *Post-hoc* Wilcoxon tests revealed that participants endorsed a significantly greater feeling of anxiety and seeking than play, care, grief, rage or lust. However, they endorsed a significantly lesser feeling of rage than play. Lastly, they also endorsed a significantly lesser feeling of lust than play, care, grief, or rage.

#### Describing participants' emotional memory retention

**Figure 3:**
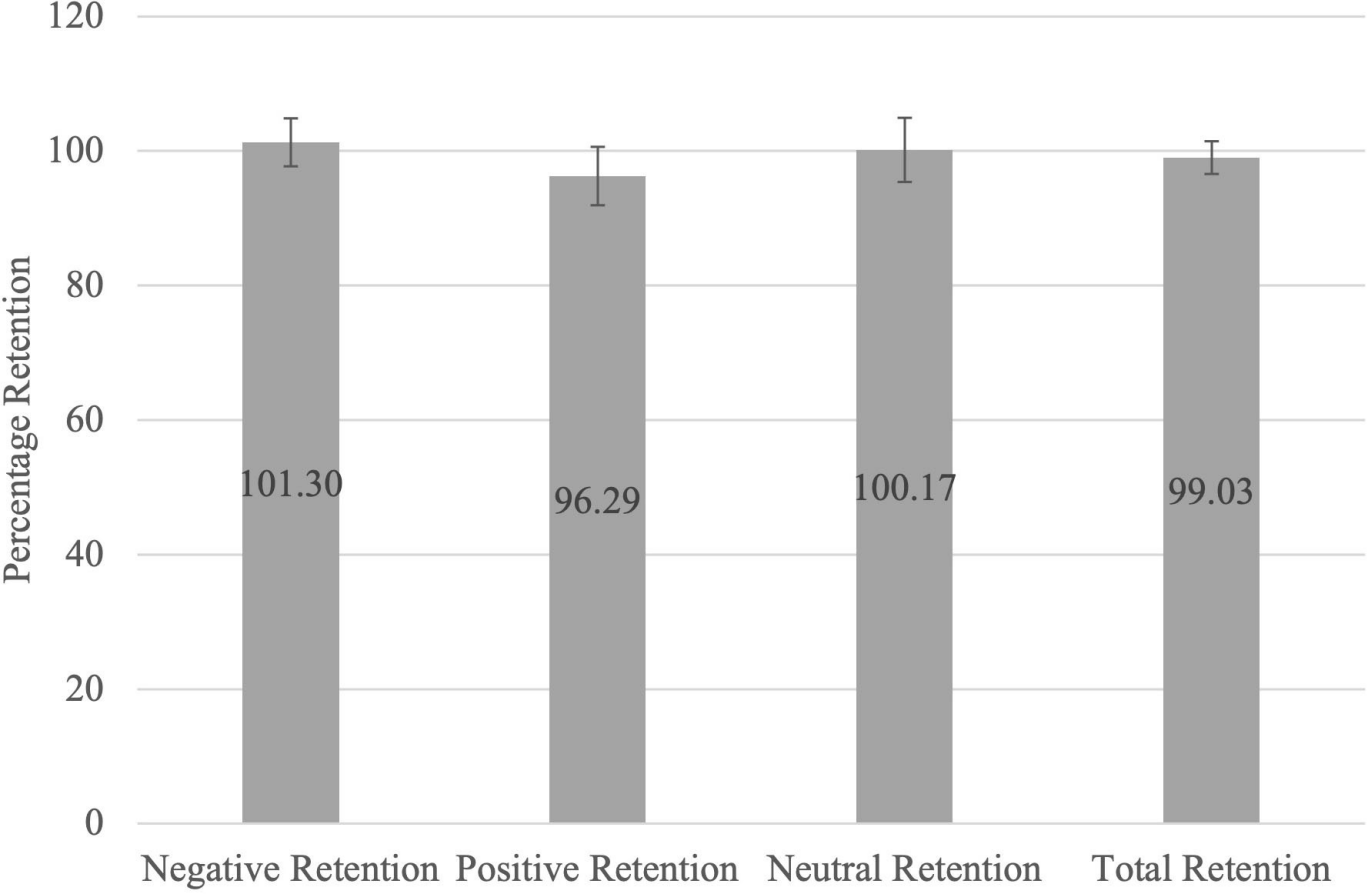
Average Memory Retention Scores for Each Valence Category. The average memory retention scores for each category (Negative, Positive, Neutral, and Total Retention) were determined by dividing the number of pictures recalled per category during the post-sleep task by the number of pictures recalled per category during the pre-sleep task and expressed as a percentage.

Regarding pre- and post-sleep memory recall, participants recalled a larger number of negative pictures than pictures in the other categories and a larger number of positive pictures than neutral pictures (Supplementary Table S1). Figure 3 shows that participants retained, across the different valence categories, what they had initially learnt. A repeated measures ANOVA determined that the differences in average memory retention for each category (positive, negative, and neutral pictures), were not significant [*F*_(2,194)_ = 2.122, *p* = 0.123, η*p*^2^ =0.021], indicating that average memory retention for all three categories was similar.

### Hypothesis testing: predicting emotional memory retention using overall dream affect intensity

**Figure 4:**
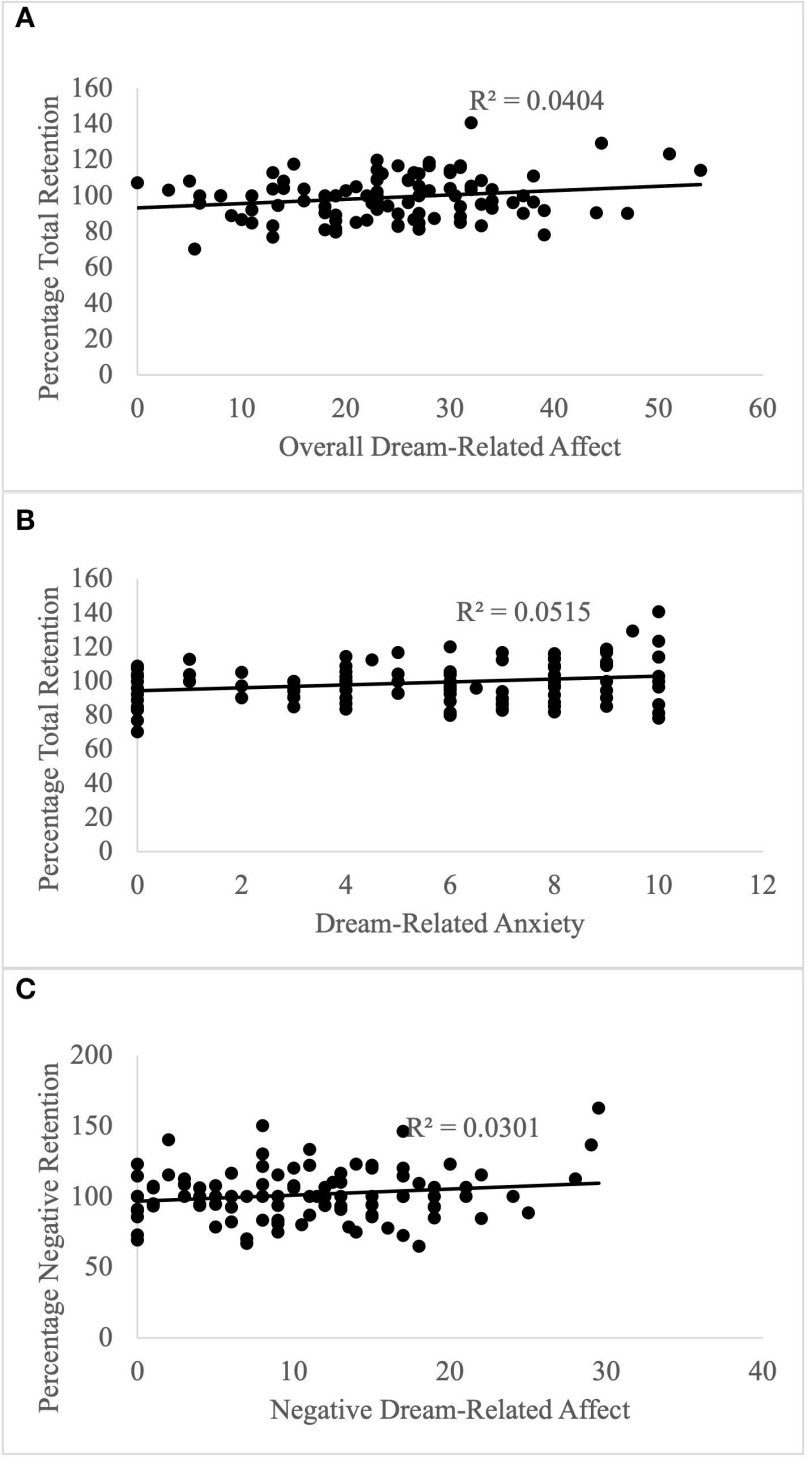
Predicting Memory Retention with Dream-Related Affect. **(A)** Overall dream-related affect predicts memory retention irrespective of valence; **(B)** Dream-related anxiety predicts memory retention irrespective of valence; **(C)** Negative dream-related affect predicts retention of negative information.

Neither average dream affect intensity nor the other control variables (age, gender, caffeine intake, smoking, and sleep quality) had any significant influence on the retention of either positive, negative or neutral information (results described in Supplementary material). However, there was a significant relationship between the retention of all information and overall dream-related affect intensity when accounting for the control variables (Table 3). As the average dream affect intensity in a dream increased, so did the retention for all information, with a small effect size (Figure 4A). Both the age of participants and their caffeine intake had a significant effect on this aspect of retention. Excluding participants who slept 6 h or less (*n* = 2), did not change the results, showing that sleep duration is unlikely to explain any significant variance in the data. The mean retention for all information was better for participants between the ages of 25 and 34 (*M* =103.4, *SD* = 16.22) and worse for younger participants, aged 18–24 (*M* = 99.8, *SD* = 12.8). Retention for all information was worse when participants had caffeine on the morning of the study (*M* = 93.57, *SD* = 10.71) compared to no caffeine on the day of the study (*M* = 100.62, *SD* = 12.44). The overall model explained 0.110 variance with a medium effect size.

**Table 3:** Predicting retention for all information using overall dream affect intensity.

**Variable**	**Type III SS**	***df***	***MS***	***F***	***p***	***ηp^2^***
Corrected model	2,116.042^a^	3	705.347	5.171	0.002	0.137
Intercept	9,367.267	1	9,367.267	68.678	< 0.001	0.412
Age	668.144	1	668.144	4.899	0.029^*^	0.048
Caffeine intake	1,110.082	1	1,110.082	8.139	0.005^**^	0.077
Total emotions	544.468	1	544.468	3.992	0.048^*^	0.039
Error	13,366.647	98	136.394			
Total	1,015,738.852	102				
Corrected Total	15,482.690	101				

* p < 0.05,

** p < 0.01. Model with the dependent variable, total retention: intercept + age + caffeine intake + total emotion.

a R^2^ = 0.137 (adjusted R^2^ = 0.110).

### Exploratory analyses: investigating the independent contributions of positive and negative dream affects on memory retention

GLM analysis showed that positive and negative affect did not differentially modulate retention for positive or neutral information (see Supplementary material). Regarding retention of negative information, dream-related negative affect predicted this component of memory retention, such that higher dream-affect was associated with better retention of negative information, with a small effect size (Table 4, Figure 4C). Regarding the influence of covariates, alertness also had a significant effect on retention, which was better when participants indicated that they felt “wide awake” upon waking (*M* = 109.82, *SD* = 20.83) compared to those who reported feeling “somewhat awake” (*M* = 97.07, *SD* = 14.65) or “still tired” (*M* = 101.05, *SD* = 19.0) upon waking. The overall model explained 0.098 variance, with a medium effect size.

**Table 4:** Predicting retention for negative information using positive and negative dream affect intensity.

**Variable**	**Type III SS**	***df***	***MS***	***F***	***p***	***ηp^2^***
Corrected model	4,097.056^a^	3	1,365.685	4.634	0.005	0.125
Intercept	296,743.847	1	296,743.847	1,006.965	< 0.001	0.912
Alertness	3,114.181	2	1,557.091	5.284	0.007^**^	0.098
Mean negative affect	1,294.618	1	1,294.618	4.393	0.039^*^	0.043
Error	28,585.045	97	294.691			
Total	1,069,091.735	101				
Corrected total	32,682.101	100				

* p < 0.05,

** p < 0.01. Model with the dependent variable, negative retention: intercept + alertness + negative emotion.

a R^2^ = 0.125 (adjusted R^2^ = 0.098).

#### Predicting emotional memory retention using individual dream affect intensity

Lastly, we ran a follow-up analysis regarding the two memory retention categories that showed an influence of dream affect (retention of negative and all information), to determine whether the significant contribution of dream-related affect already described was underpinned by a discrete category of positive or negative dream-related affect. No individual affects significantly predicted retention of negative information (see Supplementary material). However, dream-related anxiety predicted retention for all information, showing that an increase in anxiety led to better retention, with a small effect size (Table 5, Figure 4B). As described before, both the age of participants and their caffeine intake had a significant effect on retention. The overall model explained 0.124 variance, with a medium effect size.

**Table 5:** Predicting retention for all information using individual dream affect intensity.

**Variable**	**Type III SS**	***df***	***MS***	***F***	***p***	***ηp^2^***
Corrected Model	2,316.465^a^	3	772.155	5.747	0.001	0.150
Intercept	9,929.901	1	9,929.901	73.911	< 0.001	0.430
Age	682.514	1	682.514	5.080	0.026^*^	0.049
Caffeine Intake	1,129.516	1	1,129.516	8.407	0.005^**^	0.079
Anxiety	744.891	1	744.891	5.544	0.021^*^	0.054
Error	13,166.225	98	134.349			
Total	1,015,738.852	102				
Corrected Total	15,482.690	101				

* p < 0.05,

** p < 0.01. Model with the dependent variable, total retention: intercept + age + caffeine intake + anxiety.

a R^2^ = 0.150 (adjusted R^2^ = 0.124) .

## Discussion

This study aimed to determine whether affect experienced in dreams contributed to emotional memory consolidation, where affect refers to the subjective experience of emotions and where emotional memory consolidation was measured by memory retention for valenced information (i.e., positive and negative information). We predicted that the greater the intensity of affect in dreams (regardless of the valence), the greater the memory retention would be for emotional information, but not neutral information.

Our findings showed that average dream affect intensity did not predict retention of positive or negative information. However, average dream affect intensity did significantly predict memory retention for all information, indicating that as average dream affect intensity increased, so did retention. Furthermore, dream-related anxiety significantly predicted the retention of all information. Therefore, as anxiety in a dream increased, so did overall memory retention. Lastly, negative affects in dreams significantly predicted retention of negative information, where an increase in negative affects in dreams led to an increase in retention of negative information.

### The effect of dream affect intensity on memory consolidation

Both sleep and affect independently influence declarative memory (Nishida et al., 2009). An established body of literature shows that after a period of sleep, in comparison to an equivalent period of waking, there is better retention of declarative information. Separately, research findings show that affect enhances memory consolidation (Tyng et al., 2017). Therefore, if emotions are activated during dreaming, emotion activation in this context may also lead to memory consolidation enhancement. The amygdala and the mesolimbic dopaminergic pathway play a role in affect regulation and memory. These same structures and pathways are also central to dreaming (Solms, 2000; Perogamvros et al., 2013), suggesting that there is a possible common neurobiological basis underlying the role of dream-related affect in memory consolidation processes. Our findings suggest that if emotions are activated during dreaming, imbued with memory traces (generally for positive, negative, and neutral information), then this coupling of affect and memory traces may result in preferential sleep-dependent consolidation, although with a modest effect. Behavioural data has shown that those individuals who incorporate a recent learning experience into their dream content, also have better memory consolidation than those with less incorporation of the learning task (Wamsley and Stickgold, 2019). Our findings show, that in addition to the relevance of dream content, dream affect is also a contributor to memory consolidation.

While the initial results show that this effect is non-specific, that is all dream-related affect results in a general memory consolidation effect, our findings also show that the experience of anxiety in dreams had a specific effect on the consolidation of memory, irrespective of valence. Anxiety was also the most highly rated affect in dream reports. Research has shown that the experience of fear and panic, associated with anxiety, signals the release of the stress hormone, cortisol (Bandelow et al., 2000; Hannibal and Bishop, 2014). Although in our study, we are not able to determine to what extent participants experienced REM sleep, studies show that both REM sleep and cortisol secretion increase during the second half of the night (Vgontzas et al., 1997). Cortisol continues to rise during this time of the night to peak with the cortisol awakening response (Hirotsu et al., 2015). Moreover, studies have shown that cortisol promotes sleep-dependent memory consolidation (Bennion et al., 2015). Therefore, speculatively, the experience of anxiety in dreams during this part of the night, when cortisol secretion is at its highest, may have aided in the modulation of memory consolidation. An alternative explanation for our findings could be that cortisol acts as a third variable influencing both memory consolidation and dream-related anxiety. An increase in cortisol may aid in the modulation of memory consolidation and also lead to increased feelings of anxiety in dreams.

### The specific effects of negative dream affect on consolidation of negative information

While this study found a general memory consolidation effect, it also found an effect that is specific to memory retention for negative information, showing that the negative affect in dreams significantly predicted memory retention for negative information. Some previous research shows that sleep benefits the consolidation of emotionally negative over neutral memories, including two recent meta-analyses, although only under certain conditions (Lipinska et al., 2019; Schäfer et al., 2020 but see Bolinger et al., 2018 or Jones et al., 2018 for alternative findings). However, the mechanism behind this consolidation effect is unknown. Our findings suggest that negative affect during dreaming could be one key mechanism that contributes to the consolidation of negative memories. Although our study showed that all information was consolidated to a similar degree, there may be different mechanisms contributing to the consolidation of negative, positive and neutral information and for negative stimuli, dream affect may have had a significant role in this process.

More broadly, emotional memory consolidation is a necessary process that integrates new affective experiences into existing memory networks (e.g., Payne et al., 2009). This processing ensures that previous experiences and memories can be used in future to aid in decision-making and guiding behaviour. Many researchers have suggested that dreaming also supports the processing of emotions to guide future behaviour (Cartwright, 1974; van der Helm and Walker, 2011; Lipinska et al., 2019). Negative affect in dreams might, therefore, be a way of indicating which experiences are salient and require consolidation, in the process of learning from experience to update predictive models guiding behaviour.

Interestingly, the positive affect in dreams had no significant influence on memory consolidation. A possible explanation for this is that there is an asymmetry in how people use positive and negative information. People tend to show a negativity bias where negative information gets attended to, learnt from, and used, much more readily than positive information (Vaish et al., 2008). Overall, positive affect and positive information were likely not salient enough to elicit the same effects seen with negative affect and negative information. An alternative explanation for the observed findings is that the arousal component related to positive affect was lower than for negative affect. Some studies suggest that arousal, rather than valence, is the driving factor strengthening memory, and in many cases, negative emotions have a higher arousal component (Mather and Nesmith, 2008).

### Implication of findings

These findings can have implications for mood disorders such as depression, which are characterised by negative affect and increased memory sensitivity to negative stimuli (Hamilton and Gotlib, 2008). The findings of this study show that an increased experience of negative affect in dreaming leads to an increase in memory consolidation for negative information. If mood disorders, such as depression already have a predisposition to the experience of negative affect, then experiencing emotionally negative dreams can perpetuate depressive states because it leads to an increased focus on negative information. This finding is supported by research that has found that therapeutic REM sleep deprivation, which decreases the amount of time spent in REM sleep where dreaming is most commonly reported, can decrease symptoms of depression (Riemann et al., 2020). Further, most antidepressants affect sleep by suppressing REM sleep, which is a component of the mechanism of action of antidepressants (Steiger and Pawlowski, 2019; Riemann et al., 2020).

### Other influencing factors

#### Age, caffeine intake and alertness

Age had a significant effect on memory retention for all information, where older participants (aged 25–34) had better retention than younger participants (aged 18–24). This is a counterintuitive finding since typically memory performance becomes worse with age, a finding supported by multiple strands of research (e.g., Steinberg et al., 2013; Richmond et al., 2022). Notably, the effects of age were modest and the age gap between participants was not significantly large with all participants falling in the young adult category.

Morning caffeine intake before the experimental sleep night also had a convincing effect on memory retention for all information, with participants who consumed coffee retaining less information compared to those who chose to not have any caffeine on the day of the study. In contradiction to our finding, some studies suggest that caffeine intake enhances memory performance (Sherman et al., 2016; Borota et al., 2018). However, caffeine intake up to 6 h before sleep causes sleep disruption, specifically a reduction in total sleep time (Drake et al., 2013). In our study, if those participants drinking coffee did not accurately report the timing of their caffeine intake, it is possible that they had worse sleep quality and therefore sleep-dependent consolidation. However, no association between caffeine intake and sleep quality (shorter sleep duration, greater number, and duration of awakenings, longer sleep latency) or alertness was found.

Alertness had a significant effect on the retention of negative information, where those who indicated that they felt wide awake upon waking, had better memory retention for negative information. Alertness is a component of sleep quality. Therefore, feeling wide awake after sleep suggests good sleep quality, with better sleep quality associated with successful memory consolidation (Murre et al., 2014).

### Limitations and future directions

This study made use of at-home data collection to minimise risk during the COVID-19 pandemic. However, in doing so, several aspects of the data collection procedure could not be carefully controlled for, including whether participants slept at the agreed-upon times, whether their dreams were from REM or NREM sleep, whether they followed the instructions to not take in any other information after the emotional memory task, and any potential internet connectivity issues that may have influenced the memory task. All of these factors may have tempered the size of the associations between dream affect and measures of memory consolidation. Similar findings with smaller effects for home-based sleep and memory studies have been reported by other authors (e.g., Maski et al., 2015; Graveline and Wamsley, 2017). Considering these limitations, future studies can opt for a sleep laboratory setting, where additionally REM parameters can be recorded.

Furthermore, studies should investigate whether the dream-related modulation effect of emotional memory can be explained by the arousal component of emotion or is specific to certain affects or a valence category.

Lastly, dream content is known to influence memory consolidation and it is unknown to what extent the task elements may have been activated during dreaming and supported memory consolidation. Future studies should look at both dream-related content and affect in the same study to differentiate the contributions of both of these to consolidation processes.

## Summary and conclusion

This study set out to determine whether there is a relationship between the affect experienced in dreams and emotional memory consolidation. Contrary to the prediction, dream-related affect did not predict emotional memory consolidation for both positive and negative information. However, with greater dream affect intensity, and specifically greater anxiety, participants retained a greater proportion of all the pictures after sleep, irrespective of the valence of the memory task stimuli. These findings suggest that when dream-related affect is higher, there is improved sleep-dependent memory consolidation. In addition to this finding, a higher degree of negative dream affect was associated with better post-sleep memory retention for negative information, highlighting that dream affect is not only associated with a general consolidation effect but is specific to negative information. Because a high proportion of dreams emerge from REM sleep, which promotes emotional memory consolidation, dreaming could be a potential mechanism underlying the consolidation of emotional memory, with emotional memories representing salient experiences that can guide future behaviour. These findings are relevant for psychiatric disorders such as major depression, which is characterised by negative affect and increased memory sensitivity to negative stimuli.

## Data availability statement

The original contributions presented in the study are included in the article/Supplementary material, further inquiries can be directed to the corresponding author.

## Ethics statement

The studies involving humans were approved by Ethics Review Committee of the Faculty of Humanities at the University of Cape Town. The studies were conducted in accordance with the local legislation and institutional requirements. The participants provided their written informed consent to participate in this study.

## Author contributions

LP and GL contributed to the conception and design of the study, performed the statistical analysis, were involved in the interpretation of the data, wrote sections of the manuscript during the second draft, contributed to the manuscript revision and editing, read, and approved the submitted version. LP conducted the data collection, prepared the data for analysis, and wrote the first draft of the manuscript. Both authors contributed to the article and approved the submitted version.
