# Supplementary material for: The modulation of emotional memory consolidation by dream affect
Source: Front Sleep. 2023 Oct 11;2:1239530. doi: 10.3389/frsle.2023.1239530 (PMC12713797; doi:10.3389/frsle.2023.1239530)
Supplement: Supplementary file 1 [file Table_1.DOCX]

**Supplementary Materials: Selection of Pictorial Stimuli and Designing the Emotional Memory Task**

The SA-APS pictures were inserted into an Excel spreadsheet and sorted according to their valence. Pictures with valence ratings between 1 and 3.99 were placed into a new sheet, named Negative, those ranging between 4 and 5.99 were placed into a sheet named Neutral and those ranging between 6 and 9 were placed into a sheet named Positive. Each of the three new sheets was then arranged according to the arousal rating of the pictures (ordered from low to high arousal ratings). The top half of the Negative sheet was pasted into a new sheet, named Negative_Calm and the bottom half was named Negative_Aroused. The same was done for the Positive sheet. The Neutral sheet was not arranged according to arousal (Backs, Da Silva, & Han, 2005). The correct number of pictures was then chosen from each of the five spreadsheets by using the RandBetween Excel function to randomly generate row numbers. This procedure was followed to ensure a representative sample of the pictures regarding their valence and arousal properties.

The pictures corresponding to the randomly generated row numbers were then pasted into a PowerPoint presentation. The picture order was randomised but ensured that no more than four pictures from the same valence were shown consecutively (Ackermann et al., 2015). One black slide and one black slide with a white fixation cross were shown for 500ms each before a new picture. The picture was then presented for 2.5 seconds (Ackermann et al., 2015). After the participants watched the entire slideshow, they were instructed to verbally recall as many pictures as possible by describing each picture in as much detail as possible. Participants were prompted with phrases such as “Is there anything else you can remember?”. The task did not have a time limit and participants were also not told how many pictures there were.

**Supplementary Materials: Participants’ Pre- and Post-Sleep Memory Recall**

**Table 1**

*Pre- and Post-Sleep Memory Recall of Negative, Positive and Neutral Information*

| Condition | Negative | Positive | Neutral | X^2^ | df | Asymp. Sig |
| --- | --- | --- | --- | --- | --- | --- |
|  | M (SD) | M (SD) | M (SD) |  |  |  |
| Pre-Sleep | 13.14 (3.384) | 8.55 (3.096) | 7.03 (2.447) | 140.036 | 2 | <.001** |
| Contrast 1 |  |  |  | -8.263 |  | <.001** |
| Contrast 2 |  |  |  | -4.656 |  | <.001** |
| Contrast 3 |  |  |  | -8.546 |  | <.001** |
| Post-Sleep | 12.97 (3.339) | 8.18 (3.070) | 7.00 (2.730) | 134.291 | 2 | <.001** |
| Contrast 1 |  |  |  | -8.264 |  | <.001** |
| Contrast 2 |  |  |  | -4.014 |  | <.001** |
| Contrast 3 |  |  |  | -8.800 |  | <.001** |

*Note:* * p<.05, **p<.01. Contrast 1: Positive vs Negative. Contrast 2: Positive vs Neutral. Contrast 3: Negative vs Neutral.

**Supplementary Materials: Participants’ Raw Pre- and Post-Sleep Memory Performance**

**Table 2**

*Participants’ Raw Pre- and Post-Sleep Memory Performance*

| **Participant** | **Night Negative** | **Morning Negative** | **Negative Retention** | **Night Positive** | **Morning Positive** | **Positive Retention** | **Night Neutral** | **Morning Neutral** | **Neutral Retention** | **Total Retention** |
| --- | --- | --- | --- | --- | --- | --- | --- | --- | --- | --- |
| 1 | 10 | 7 | 70.00 | 9 | 8 | 88.89 | 5 | 5 | 100.00 | 83.33 |
| 2 | 10 | 11 | 110.00 | 10 | 11 | 110.00 | 4 | 5 | 125.00 | 112.50 |
| 3 | 10 | 12 | 120.00 | 6 | 5 | 83.33 | 8 | 10 | 125.00 | 112.50 |
| 4 | 12 | 12 | 100.00 | 10 | 9 | 90.00 | 7 | 7 | 100.00 | 96.55 |
| 5 | 12 | 13 | 108.33 | 7 | 7 | 100.00 | 7 | 7 | 100.00 | 103.85 |
| 6 | 15 | 13 | 86.67 | 8 | 6 | 75.00 | 6 | 6 | 100.00 | 86.21 |
| 7 | 18 | 21 | 116.67 | 5 | 7 | 140.00 | 9 | 10 | 111.11 | 118.75 |
| 8 | 18 | 18 | 100.00 | 14 | 13 | 92.86 | 10 | 10 | 100.00 | 97.62 |
| 9 | 13 | 12 | 92.31 | 4 | 5 | 125.00 | 7 | 8 | 114.29 | 104.17 |
| 10 | 13 | 15 | 115.38 | 7 | 5 | 71.43 | 8 | 7 | 87.50 | 96.43 |
| 11 | 18 | 14 | 77.78 | 13 | 11 | 84.62 | 10 | 9 | 90.00 | 82.93 |
| 12 | 10 | 12 | 120.00 | 5 | 4 | 80.00 | 5 | 3 | 60.00 | 95.00 |
| 13 | 16 | 15 | 93.75 | 7 | 8 | 114.29 | 9 | 9 | 100.00 | 100.00 |
| 14 | 13 | 16 | 123.08 | 10 | 13 | 130.00 | 4 | 9 |  | 140.74 |
| 15 | 13 | 15 | 115.38 | 11 | 11 | 100.00 | 4 | 7 | 175.00 | 117.86 |
| 16 | 14 | 11 | 78.57 | 7 | 9 | 128.57 | 7 | 6 | 85.71 | 92.86 |
| 17 | 15 | 15 | 100.00 | 9 | 9 | 100.00 | 6 | 6 | 100.00 | 100.00 |
| 18 | 13 | 14 | 107.69 | 7 | 6 | 85.71 | 7 | 8 | 114.29 | 103.70 |
| 19 | 17 | 15 | 88.24 | 14 | 12 | 85.71 | 10 | 10 | 100.00 | 90.24 |
| 20 | 17 | 16 | 94.12 | 15 | 16 | 106.67 | 11 | 8 | 72.73 | 93.02 |
| 21 | 14 | 17 | 121.43 | 14 | 12 | 85.71 | 6 | 8 | 133.33 | 108.82 |
| 22 | 10 | 13 | 130.00 | 9 | 8 | 88.89 | 5 | 5 | 100.00 | 108.33 |
| 23 | 11 | 10 | 90.91 | 5 | 6 | 120.00 | 4 | 3 | 75.00 | 95.00 |
| 24 | 13 | 12 | 92.31 | 13 | 9 | 69.23 | 6 | 8 | 133.33 | 90.63 |
| 25 | 10 | 11 | 110.00 | 7 | 8 | 114.29 | 6 | 6 | 100.00 | 108.70 |
| 26 | 15 | 16 | 106.67 | 10 | 9 | 90.00 | 10 | 9 | 90.00 | 97.14 |
| 27 | 15 | 14 | 93.33 | 9 | 11 | 122.22 | 8 | 8 | 100.00 | 103.13 |
| 28 | 10 | 9 | 90.00 | 7 | 10 | 142.86 | 7 | 7 | 100.00 | 108.33 |
| 29 | 14 | 12 | 85.71 | 5 | 3 | 60.00 | 7 | 5 | 71.43 | 76.92 |
| 30 | 16 | 15 | 93.75 | 6 | 5 | 83.33 | 6 | 5 | 83.33 | 89.29 |
| 31 | 15 | 15 | 100.00 | 9 | 10 | 111.11 | 10 | 7 | 70.00 | 94.12 |
| 32 | 11 | 11 | 100.00 | 4 | 4 | 100.00 | 3 | 4 | 133.33 | 105.56 |
| 33 | 9 | 12 | 133.33 | 8 | 7 | 87.50 | 7 | 9 | 128.57 | 116.67 |
| 34 | 20 | 15 | 75.00 | 16 | 13 | 81.25 | 12 | 14 | 116.67 | 87.50 |
| 35 | 18 | 15 | 83.33 | 14 | 10 | 71.43 | 11 | 11 | 100.00 | 83.72 |
| 36 | 13 | 19 | 146.15 | 11 | 11 | 100.00 | 11 | 10 | 90.91 | 114.29 |
| 37 | 15 | 13 | 86.67 | 10 | 9 | 90.00 | 6 | 6 | 100.00 | 90.32 |
| 38 | 14 | 12 | 85.71 | 11 | 10 | 90.91 | 6 | 6 | 100.00 | 90.32 |
| 39 | 13 | 16 | 123.08 | 12 | 10 | 83.33 | 13 | 9 | 69.23 | 92.11 |
| 40 | 8 | 9 | 112.50 | 5 | 3 | 60.00 | 7 | 5 | 71.43 | 85.00 |
| 41 | 20 | 17 | 85.00 | 8 | 10 | 125.00 | 9 | 9 | 100.00 | 97.30 |
| 42 | 6 | 9 | 150.00 | 7 | 7 | 100.00 | 6 | 4 | 66.67 | 105.26 |
| 43 | 14 | 16 | 114.29 | 12 | 12 | 100.00 | 10 | 6 | 60.00 | 94.44 |
| 44 | 17 | 18 | 105.88 | 13 | 14 | 107.69 | 11 | 15 | 136.36 | 114.63 |
| 45 | 14 | 16 | 114.29 | 11 | 12 | 109.09 | 11 | 9 | 81.82 | 102.78 |
| 46 | 13 | 14 | 107.69 | 10 | 6 | 60.00 | 4 | 4 | 100.00 | 88.89 |
| 47 | 18 | 17 | 94.44 | 5 | 8 | 160.00 | 7 | 10 | 142.86 | 116.67 |
| 48 | 10 | 10 | 100.00 | 6 | 7 | 116.67 | 7 | 8 | 114.29 | 108.70 |
| 49 | 18 | 17 | 94.44 | 10 | 10 | 100.00 | 11 | 12 | 109.09 | 100.00 |
| 50 | 16 | 17 | 106.25 | 15 | 16 | 106.67 | 13 | 15 | 115.38 | 109.09 |
| 51 | 17 | 11 | 64.71 | 7 | 7 | 100.00 | 8 | 8 | 100.00 | 81.25 |
| 52 | 9 | 9 | 100.00 | 7 | 7 | 100.00 | 5 | 4 | 80.00 | 95.24 |
| 53 | 11 | 11 | 100.00 | 9 | 10 | 111.11 | 9 | 8 | 88.89 | 100.00 |
| 54 | 9 | 11 | 122.22 | 9 | 10 | 111.11 | 7 | 8 | 114.29 | 116.00 |
| 55 | 15 | 15 | 100.00 | 6 | 6 | 100.00 | 6 | 8 | 133.33 | 107.41 |
| 56 | 13 | 15 | 115.38 | 7 | 6 | 85.71 | 6 | 6 | 100.00 | 103.85 |
| 57 | 14 | 15 | 107.14 | 7 | 5 | 71.43 | 4 | 5 | 125.00 | 100.00 |
| 58 | 15 | 16 | 106.67 | 14 | 15 | 107.14 | 7 | 6 | 85.71 | 102.78 |
| 59 | 5 | 7 | 140.00 | 7 | 7 | 100.00 | 5 | 3 | 60.00 | 100.00 |
| 60 | 8 | 13 | 162.50 | 3 | 4 | 133.33 | 6 | 5 | 83.33 | 129.41 |
| 61 | 17 | 18 | 105.88 | 14 | 14 | 100.00 | 11 | 11 | 100.00 | 102.38 |
| 62 | 14 | 11 | 78.57 | 10 | 9 | 90.00 | 10 | 10 | 100.00 | 88.24 |
| 63 | 19 | 18 | 94.74 | 4 | 8 |  | 8 | 9 | 112.50 | 112.90 |
| 64 | 9 | 11 | 122.22 | 7 | 6 | 85.71 | 5 | 4 | 80.00 | 100.00 |
| 65 | 12 | 10 | 83.33 | 9 | 7 | 77.78 | 1 | 1 | 100.00 | 81.82 |
| 66 | 19 | 19 | 100.00 | 11 | 12 | 109.09 | 6 | 7 | 116.67 | 105.56 |
| 67 | 12 | 12 | 100.00 | 9 | 7 | 77.78 | 6 | 4 | 66.67 | 85.19 |
| 68 | 10 | 12 | 120.00 | 3 | 6 |  | 10 | 8 | 80.00 | 113.04 |
| 69 | 15 | 16 | 106.67 | 10 | 11 | 110.00 | 2 | 3 | 150.00 | 111.11 |
| 70 | 12 | 1 |  | 6 | 6 | 100.00 | 6 | 2 | 33.33 |  |
| 71 | 11 | 15 | 136.36 | 14 | 13 | 92.86 | 10 | 12 | 120.00 | 114.29 |
| 72 | 16 | 15 | 93.75 | 12 | 10 | 83.33 | 5 | 6 | 120.00 | 93.94 |
| 73 | 4 | 7 |  | 7 | 7 | 100.00 | 9 | 6 | 66.67 | 100.00 |
| 74 | 11 | 9 | 81.82 | 7 | 6 | 85.71 | 5 | 5 | 100.00 | 86.96 |
| 75 | 12 | 12 | 100.00 | 9 | 10 | 111.11 | 6 | 4 | 66.67 | 96.30 |
| 76 | 13 | 11 | 84.62 | 6 | 4 | 66.67 | 4 | 3 | 75.00 | 78.26 |
| 77 | 16 | 17 | 106.25 | 10 | 11 | 110.00 | 8 | 9 | 112.50 | 108.82 |
| 78 | 4 | 4 | 100.00 | 4 | 4 | 100.00 | 5 | 5 | 100.00 | 100.00 |
| 79 | 12 | 9 | 75.00 | 9 | 8 | 88.89 | 6 | 6 | 100.00 | 85.19 |
| 80 | 16 | 15 | 93.75 | 10 | 10 | 100.00 | 12 | 11 | 91.67 | 94.74 |
| 81 | 11 | 11 | 100.00 | 7 | 7 | 100.00 | 5 | 8 | 160.00 | 113.04 |
| 82 | 11 | 10 | 90.91 | 7 | 5 | 71.43 | 6 | 5 | 83.33 | 83.33 |
| 83 | 15 | 15 | 100.00 | 9 | 5 | 55.56 | 6 | 6 | 100.00 | 86.67 |
| 84 | 18 | 13 | 72.22 | 12 | 10 | 83.33 | 8 | 8 | 100.00 | 81.58 |
| 85 | 14 | 14 | 100.00 | 8 | 9 | 112.50 | 7 | 7 | 100.00 | 103.45 |
| 86 | 11 | 8 | 72.73 | 11 | 7 | 63.64 | 5 | 4 | 80.00 | 70.37 |
| 87 | 21 | 17 | 80.95 | 11 | 9 | 81.82 | 10 | 12 | 120.00 | 90.48 |
| 88 | 10 | 8 | 80.00 | 8 | 9 | 112.50 | 7 | 7 | 100.00 | 96.00 |
| 89 | 14 | 14 | 100.00 | 2 | 2 | 100.00 | 8 | 8 | 100.00 | 100.00 |
| 90 | 11 | 12 | 109.09 | 7 | 5 | 71.43 | 6 | 8 | 133.33 | 104.17 |
| 91 | 8 | 9 | 112.50 | 4 | 7 | 175.00 | 5 | 5 | 100.00 | 123.53 |
| 92 | 15 | 10 | 66.67 | 8 | 11 | 137.50 | 7 | 6 | 85.71 | 90.00 |
| 93 | 14 | 13 | 92.86 | 4 | 3 | 75.00 | 7 | 7 | 100.00 | 92.00 |
| 94 | 12 | 13 | 108.33 | 8 | 8 | 100.00 | 5 | 9 | 180.00 | 120.00 |
| 95 | 10 | 10 | 100.00 | 7 | 6 | 85.71 | 7 | 7 | 100.00 | 95.83 |
| 96 | 15 | 14 | 93.33 | 8 | 7 | 87.50 | 7 | 3 | 42.86 | 80.00 |
| 97 | 12 | 12 | 100.00 | 4 | 4 | 100.00 | 3 | 3 | 100.00 | 100.00 |
| 98 | 13 | 16 | 123.08 | 6 | 7 | 116.67 | 5 | 5 | 100.00 | 116.67 |
| 99 | 11 | 11 | 100.00 | 9 | 6 | 66.67 | 2 | 2 | 100.00 | 86.36 |
| 100 | 12 | 14 | 116.67 | 9 | 5 | 55.56 | 6 | 6 | 100.00 | 92.59 |
| 101 | 13 | 9 | 69.23 | 13 | 12 | 92.31 | 7 | 7 | 100.00 | 84.85 |
| 102 | 14 | 14 | 100.00 | 5 | 5 | 100.00 | 7 | 7 | 100.00 | 100.00 |
| 103 | 11 | 10 | 90.91 | 7 | 3 | 42.86 | 6 | 7 | 116.67 | 83.33 |

*Note.* The missing data points have been excluded due to being extreme outliers.

Supplementary Materials: Predicting Retention of Positive, Negative and Neutral Information Using Overall Dream Affect Intensity

**Table 3**

*Predicting Retention of Positive Information Using Overall* *Dream Affect Intensity*

| Variable | *Type III SS* | *df* | *MS* | *F* | *p* | *ηp ^2^* |
| --- | --- | --- | --- | --- | --- | --- |
| Corrected Model | 2531.566^a^ | 5 | 506.313 | 1.049 | .394 | .052 |
| Intercept | 8741.123 | 1 | 8741.123 | 18.116 | <.001 | .160 |
| Age | 581.372 | 1 | 581.372 | 1.205 | .275 | .013 |
| Gender | 36.263 | 1 | 36.263 | .075 | .785 | .001 |
| Caffeine Intake | 1132.434 | 1 | 1132.434 | 2.347 | .129 | .024 |
| Smoking | 286.937 | 1 | 286.937 | .595 | .443 | .006 |
| Total Emotions | 556.269 | 1 | 556.269 | 1.153 | .286 | .012 |
| Error | 45839.271 | 95 | 482.519 |  |  |  |
| Total | 984762.923 | 101 |  |  |  |  |
| Corrected Total | 48370.837 | 100 |  |  |  |  |

*Note:* * p<.05, **p<.01. Model with the dependent variable, Positive Retention: Intercept + Age + Gender + Caffeine Intake + Smoking + Total Emotion.

^a.^ R Squared = .052 (Adjusted R Squared = .002)

This model indicates that there is no significant relationship between the retention of positive information and overall dream affect intensity when accounting for all the control variables. Further, none of the control variables had a significant effect on the retention of positive information. The overall model explained .002 variance, with a small effect size.

**Table 4**

*Predicting Retention of Negative Information Using Overall Dream Affect Intensity*

| Variable | *Type III SS* | *df* | *MS* | F | *p* | ηp ^2^ |
| --- | --- | --- | --- | --- | --- | --- |
| Corrected Model | 4664.719^a^ | 4 | 1166.180 | 3.996 | .005 | .143 |
| Intercept | 10223.024 | 1 | 10223.024 | 35.029 | <.001 | .267 |
| Age | 998.889 | 1 | 998.889 | 3.423 | .067 | .034 |
| Alertness | 2844.028 | 2 | 1422.014 | 4.872 | .010** | .092 |
| Total Emotions | 820.445 | 1 | 820.445 | 2.811 | .097 | .028 |
| Error | 28017.382 | 96 | 291.848 |  |  |  |
| Total | 1069091.735 | 101 |  |  |  |  |
| Corrected Total | 32682.101 | 100 |  |  |  |  |

*Note:* * p<.05, **p<.01. Model with the dependent variable, Negative Retention: Intercept + Age + Alertness + Total Emotion.

^a.^ R Squared = .143 (Adjusted R Squared = .107)

**Table 5**

*Predicting Retention of Neutral Information Using Overall* *Dream Affect Intensity*

| Variable | *Type III SS* | *df* | *MS* | *F* | *p* | *ηp ^2^* |
| --- | --- | --- | --- | --- | --- | --- |
| Corrected Model | 2802.119^a^ | 5 | 560.424 | .948 | .454 | .047 |
| Intercept | 13871.154 | 1 | 13871.154 | 23.458 | <.001 | .196 |
| Age | 78.434 | 1 | 78.434 | .133 | .717 | .001 |
| Gender | 371.540 | 1 | 371.540 | .628 | .430 | .007 |
| Caffeine Intake | 666.955 | 1 | 666.955 | 1.128 | .291 | .012 |
| Smoking | 987.057 | 1 | 987.057 | 1.669 | .199 | .017 |
| Total Emotions | 623.303 | 1 | 623.303 | 1.054 | .307 | .011 |
| Error | 56766.006 | 96 | 591.313 |  |  |  |
| Total | 1083128.502 | 102 |  |  |  |  |
| Corrected Total | 59568.125 | 101 |  |  |  |  |

*Note:* * p<.05, **p<.01. Model with the dependent variable, Neutral Retention: Intercept + Age + Gender + Caffeine Intake + Smoking + Total Emotion.

^a.^ R Squared = .047 (Adjusted R Squared = -.003)

This model indicates that there is no significant relationship between the retention of neutral information and overall dream affect intensity when accounting for all the control variables. Further, none of the control variables had a significant effect on the retention of neutral information. The overall model explained -.003 variance, with a small effect size.

Supplementary Materials: Predicting Retention of Positive and Neutral Information Using Positive and Negative Dream Affect Intensity

**Table 6**

*Predicting Retention of Positive Information Using Positive and Negative* *Dream Affect Intensity*

| Variable | *Type III SS* | *df* | *MS* | *F* | *p* | *ηp ^2^* |
| --- | --- | --- | --- | --- | --- | --- |
| Corrected Model | 2739.491^a^ | 6 | 456.582 | .941 | .470 | .057 |
| Intercept | 8944.411 | 1 | 8944.411 | 18.425 | <.001 | .164 |
| Age | 535.167 | 1 | 535.167 | 1.102 | .296 | .012 |
| Gender | 25.150 | 1 | 25.150 | .052 | .820 | .001 |
| Caffeine Intake | 1024.069 | 1 | 1024.069 | 2.110 | .150 | .022 |
| Smoking | 315.999 | 1 | 315.999 | .651 | .422 | .007 |
| Negative Emotions | 704.836 | 1 | 704.836 | 1.452 | .231 | .015 |
| Positive Emotions | 3.072 | 1 | 3.072 | .006 | .937 | .000 |
| Error | 45631.346 | 94 | 485.440 |  |  |  |
| Total | 984762.923 | 101 |  |  |  |  |
| Corrected Total | 48370.837 | 100 |  |  |  |  |

*Note:* * p<.05, **p<.01. Model with the dependent variable, Positive Retention: Intercept + Age + Gender + Caffeine Intake + Smoking + Negative Emotion + Positive Emotion.

^a.^ R Squared = .057 (Adjusted R Squared = -.004)

This model indicates that there is no significant relationship between the retention of positive information and dream-related positive or negative affect when accounting for all the control variables. Further, none of the control variables had a significant effect on the retention of positive information. The overall model explained -.004 variance, with a small effect size.

**Table 7**

*Predicting Retention of Neutral Information Using Positive and Negative* *Dream Affect Intensity*

| Variable | *Type III SS* | *df* | *MS* | *F* | *p* | *ηp ^2^* |
| --- | --- | --- | --- | --- | --- | --- |
| Corrected Model | 3342.187^a^ | 6 | 557.031 | .941 | .469 | .056 |
| Intercept | 12678.036 | 1 | 12678.036 | 21.421 | <.001 | .184 |
| Age | 58.771 | 1 | 58.771 | .099 | .753 | .001 |
| Gender | 299.640 | 1 | 299.640 | .506 | .478 | .005 |
| Caffeine Intake | 748.774 | 1 | 748.774 | 1.265 | .264 | .013 |
| Smoking | 1063.172 | 1 | 1063.172 | 1.796 | .183 | .019 |
| Negative Emotions | .056 | 1 | .056 | .000 | .992 | .000 |
| Positive Emotions | 1112.801 | 1 | 1112.801 | 1.880 | .174 | .019 |
| Error | 56225.938 | 95 | 591.852 |  |  |  |
| Total | 1083128.502 | 102 |  |  |  |  |
| Corrected Total | 59568.125 | 101 |  |  |  |  |

*Note:* * p<.05, **p<.01. Model with the dependent variable, Neutral Retention: Intercept + Age + Gender + Caffeine Intake + Smoking + Negative Emotion + Positive Emotion.

^a.^ R Squared = .056 (Adjusted R Squared = -.004)

This model indicates that there is no significant relationship between the retention of neutral information and dream-related positive or negative affect when accounting for all the control variables. Further, none of the control variables had a significant effect on the retention of neutral information. The overall model explained -.004 variance, with a small effect size.

Supplementary Materials: Predicting Retention of Negative Information Using Individual Dream Affect Intensity

**Table 8**

*Predicting Retention of Negative Information Using Individual Dream Affect Intensity*

| Variable | *Type III SS* | *df* | *MS* | *F* | *p* | *ηp ^2^* |
| --- | --- | --- | --- | --- | --- | --- |
| Corrected Model | 5965.745^a^ | 6 | 994.291 | 3.498 | .004 | .183 |
| Intercept | 10299.236 | 1 | 10299.236 | 36.237 | <.001 | .278 |
| Age | 1189.404 | 1 | 1189.404 | 4.185 | .044* | .043 |
| Alertness | 3298.244 | 2 | 1649.122 | 5.802 | .004** | .110 |
| Anxiety | 568.225 | 1 | 568.225 | 1.999 | .161 | .021 |
| Rage | 233.543 | 1 | 233.543 | .822 | .367 | .009 |
| Grief | 721.463 | 1 | 721.463 | 2.538 | .114 | .026 |
| Error | 26716.356 | 94 | 284.217 |  |  |  |
| Total | 1069091.735 | 101 |  |  |  |  |
| Corrected Total | 32682.101 | 100 |  |  |  |  |

*Note:* * p<.05, **p<.01. Model with the dependent variable, Negative Retention: Intercept + Age + Alertness + Anxiety + Rage + Grief.

^a.^ R Squared = .183 (Adjusted R Squared = .130)

This model indicates that there is no significant relationship between the retention of negative information and individual affects when accounting for all the control variables. Further, both Age and Alertness had a significant effect on the retention of negative information. The relationships between the retention of negative information and Age and Alertness were previously explained. The overall model explained .130 variance, with a medium effect size.
